# Family caregivers’ involvement in home-based recovery for patients with schizophrenia: a qualitative study in Beijing, China

**DOI:** 10.3389/fpsyt.2026.1853599

**Published:** 2026-07-13

**Authors:** Ting Li, Gongbo Wei, Zhaolu Pan, Dan Zhang, Meirong Wang, Lifen Chen, Guanghui Jin, Xiaoqin Lu

**Affiliations:** 1 School of General Practice and Continuing Education, Capital Medical University, Beijing, China; 2 Department of Clinical Psychology, Beijing Chao-Yang Hospital, Capital Medical University, Beijing, China; 3 Department of General Practice, Beijing Tongren Hospital, Capital Medical University, Beijing, China; 4 Department of General Practice, Beijing Pinggu Hospital, Beijing, China; 5 Department of General Practice, Beijing Chao-Yang Hospital, Capital Medical University, Beijing, China; 6 Department of Education, Xuanwu Hospital, Capital Medical University, Beijing, China

**Keywords:** family caregivers, home, involvement, recovery, schizophrenia

## Abstract

**Background:**

Family caregivers play critical roles in supporting the home-based recovery of patients with schizophrenia, but they often encounter substantial challenges and receive insufficient systemic support. Understanding caregivers’ involvement in home-based recovery is essential for aligning community mental health services with families’ capacities and needs. This study aimed to explore family caregivers’ involvement and adaptive processes in supporting home-based recovery of patients with schizophrenia in China.

**Methods:**

A qualitative study using interpretative phenomenological analysis was conducted through semi-structured interviews. Family caregivers were purposively recruited from four community health service centers (CHSCs) across urban and rural areas of Beijing. All interviews were audio-recorded, transcribed verbatim, anonymized, and analyzed iteratively to identify themes and subthemes.

**Results:**

A total of 20 family caregivers were recruited, including 11 from two urban districts and 9 from a rural district in Beijing. Five themes were identified: Caregivers’ redefinition of recovery as stability rather than cure; Routine recovery involvement in medication management and symptom monitoring; Experienced tensions between the patient’s independence and relapse prevention; Bearing family obligation and personal strain in sustained caregiving involvement; and Uncertainty in sustaining caregiving and the patients’ future stability. Caregivers reported persistent challenges in supporting patients’ independent living, participation in family activities, communication, and social interaction.

**Conclusion:**

Family caregivers gradually develop their capacity to support home-based recovery, but continue to encounter complex challenges while receiving limited support from CHSCs. Strengthening recovery-oriented family support within community mental health services, particularly through accessible psychoeducation and rehabilitation guidance, may enhance caregivers’ capacity to support patients’ independence and social participation, thereby promoting sustainable home-based care and long-term functional recovery.

## 1 Introduction

Driven by the global transition toward community-based mental health care, mental health recovery is increasingly delivered within community and home settings (1). The World Health Organization (WHO) advocates a recovery-oriented and human rights-based approach that enables individuals to regain their own identity and live meaningful, self-directed lives (2). Achieving this goal requires comprehensive community-based mental health services (1, 3). In China, efforts have been made to develop an integrated mental health service system involving mental health care institutions, community rehabilitation institutions, community-based organizations, and peer support networks (4), which promote community integration, family involvement, and home-based care (5). However, community-based mental health rehabilitation services are underdeveloped in capacity, they are yet unable to fill the gap between policy goals and actual service delivery in China (6, 7).

Due to the existing gap in community mental health services, family caregivers have taken pivotal roles increasingly to support the home-based recovery of patients with schizophrenia. WHO recommendations and international mental health practice guidelines advocate the active involvement of family caregivers in long-term recovery and community-based care (1, 8–10). Effective family involvement can improve patients’ illness management, promote functional recovery, reduce relapse risk, and alleviate caregiver’s burden (11–16). However, family caregivers’ involvement remains largely confined to daily care, emotional support, medication supervision, and symptom monitoring, while their involvement in fostering independent living skills and facilitating family communication and patients’ social interaction remains limited and inconsistent (11–17). Family caregivers often face substantial challenges in supporting patients’ recovery at home. Prolonged caregiving burden, patients’ recurrent symptoms, strained family relationships, social stigma, and limited access to professional support, all of which may compromise the quality and sustainability of home-based recovery support (18–27). In addition, many caregivers lack practical recovery-oriented caregiving knowledge and skills, including effective family communication, problem-solving skills, and medication management (28–34). Family-based interventions have demonstrated their effectiveness in preventing relapse, but most existing programs focused on psychoeducation, with scarce attention to the patients’ social rehabilitation and skill development (13–16). Furthermore, structured family interventions are poorly integrated into routine mental health services, constraining the sustainability of rehabilitative support (22, 35–37).

A deeper understanding of family caregivers’ involvement in the patients’ home-based recovery is essential to inform and better align mental health services in primary care with families’ capacities and needs. However, empirical understanding remains limited regarding family caregivers’ perspectives on their involvement in supporting the patients’ home-based recovery and the adaptive efforts they make in this process. Furthermore, family caregiving practices are embedded within broader sociocultural contexts (38). In China, where familism is deeply rooted in social norms, caregiving is widely regarded as a moral and familial obligation. This may shape how caregivers perceive their responsibilities and adapt their practices in supporting patients’ recovery at home. Therefore, an in-depth qualitative inquiry was carried out to explore the caregivers’ involvement in home-based recovery among families affected by schizophrenia in Beijing, China. The study aims to inform the development of contextually responsive, family-centered rehabilitation strategies for mental health services.

## 2 Methods

### 2.1 Study design

This qualitative study adopted interpretive phenomenological analysis (IPA) and semi-structured interviews to explore family caregivers’ involvement in supporting home-based recovery among patients with schizophrenia. Semi-structured interviews provide a flexible yet systematic approach to data collection. It enables participants to articulate their personal experiences while allowing researchers to further explore emerging perspectives and meanings in depth. IPA was used to develop a rich interpretative account of caregivers’ illness management experiences. This approach enables an in-depth exploration of how participants made sense of their lived experiences through a double hermeneutic process (39).

The interview guide was developed based on the study objectives and informed by the Family Management Style Framework (40). This framework emphasizes the integration of illness management into everyday family life through adaptive strategies and foregrounds family members’ perspectives on their roles and key components of illness management. It was originally developed in research on families of children with chronic illnesses (41) and was later extended to families of adults with conditions such as dementia and brain tumors (40, 42, 43).

### 2.2 Setting and participants

This study was carried out from September to November 2024. It was conducted at community health service center (CHSC) teaching sites affiliated with the Capital Medical University. There are 26 CHSC teaching sites spanning 13 districts across four functional areas in Beijing (comprising six urban and ten rural districts): Dongcheng and Xicheng (capital functional core area); Chaoyang, Haidian, and Fengtai (central urban area); Tongzhou, Daxing, Fangshan, and Changping (plain new town area); Pinggu, Huairou, Miyun, and Yanqing (ecological conservation area).

A three-stage sampling strategy was applied. First, one district was randomly selected from each of the three functional areas: Xicheng (capital functional core area), Fengtai (central urban area), and Pinggu (ecological conservation area). Then, four CHSCs were selected, including two from urban districts (Xicheng and Fengtai) and two from a rural district (Pinggu). Patients with schizophrenia and their primary family caregivers were recruited from the selected CHSCs.

A purposive sampling strategy was employed with assistance from mental health physicians at participating CHSCs. These physicians assisted in identifying and approaching eligible primary caregivers of patients with schizophrenia who had been diagnosed at psychiatric specialty hospitals and were receiving community-based follow-up care. The inclusion criteria of caregivers were as follows (1): aged 18 years or older; (2) live with a family member diagnosed with schizophrenia as a spouse, parent, sibling, or offspring; (3) take primary responsibility for the patient’s daily care for at least one year and are familiar with the patient’s condition and family caregiving circumstances; and (4) willing to share their caregiving experiences related to schizophrenia. Twenty-two eligible family caregivers were approached, and 20 agreed to participate in the study. Two caregivers declined participation due to stigma-related concerns and scheduling conflicts.

### 2.3 Interview guide development

Two interviewers received training in qualitative interviewing techniques prior to data collection. During a pilot phase, preliminary interviews were conducted with three participants to assess the clarity and relevance of the interview questions. Insights from these interviews informed refinement of the original interview guide, resulting in the final version used for formal data collection. The final interview guide (see **Supplementary Material**) explored family caregivers’ experiences of supporting home-based recovery among patients with schizophrenia, with particular attention to the challenges encountered and the adaptive strategies developed within the primary healthcare system.

### 2.4 Data collection

To ensure confidentiality and privacy, all interviews were conducted in soundproof private rooms. Before each interview, participants were informed of the study objectives and assured that their personal information would be kept confidential. Written informed consent was obtained from all participants. With consents, all interviews were conducted in Chinese, audio-recorded, and supplemented with field notes taken during the interview process. Each interview lasted approximately 45–60 minutes. Field notes were returned to participants for comments and corrections to enhance accuracy. Following each interview, audio recordings were transcribed verbatim and subsequently translated into English by bilingual researchers. To ensure semantic accuracy and preserve the cultural meanings embedded in participants’ accounts, translated transcripts and quotations were cross-checked against the original Chinese transcripts and discussed among the research team when necessary. After each interview, participants received a gift (a vacuum flask) as compensation for their time.

Data saturation was assessed through concurrent data collection and iterative analysis, consistent with the idiographic and interpretative orientation of IPA. After each interview, the research team reviewed transcripts, emerging themes, and reflexive notes to evaluate whether additional interviews contributed new experiential insights or meaningful variations in caregivers’ accounts. Saturation was considered achieved when no substantial new interpretative themes or variations in participants’ experiences emerged across consecutive interviews, and the thematic structure was sufficiently rich to address the research aim. This point was reached after eight caregiver interviews in urban districts and seven in the rural district. To further confirm the adequacy and depth of the thematic structure, additional interviews were conducted, resulting in a final sample of 11 caregivers in urban districts (U1-U11) and 9 caregivers in the rural district (R1-R9).

### 2.5 Data analysis

All audio-recorded interviews were transcribed verbatim within 24 hours of completion. NVivo 12 software was used for data management and coding. Data analysis followed established IPA guideline (44) and was conducted iteratively and interpretatively on a case-by-case basis before patterns across cases were explored. First, researchers repeatedly read each transcript to achieve immersion and recorded exploratory notes focusing on descriptive content, language use, emotional expressions, and conceptual reflections. Line-by-line coding was then performed to identify significant statements and experiential meanings. Interpretative memos were written throughout the process to capture reflections on how participants understood and negotiated caregiving experiences. Emergent themes were developed within each case by clustering related codes and continuously comparing themes with the original transcripts to ensure interpretations remained grounded in participants’ accounts. Subsequently, patterns of convergence and divergence across cases were explored, and recurrent themes were integrated into higher-order thematic clusters while preserving individual nuances.

Two researchers (TL, GW), both experienced in qualitative and mental health research, independently reviewed the transcripts, conducted coding, and developed themes and subthemes. Discrepancies were resolved through discussion to achieve consensus. All extracted codes and thematic categories were subsequently reviewed and approved by senior members of the research team. Given the interpretative nature of IPA, reflexive memos and regular team discussions were used throughout data collection and analysis to minimize potential interpretative bias.

### 2.6 Trustworthiness

To enhance the trustworthiness and rigor of the study, we adhered to the four established criteria of credibility, dependability, confirmability, and transferability (45, 46). Credibility was strengthened through prolonged engagement with the data, member checking, and comparison of accounts across caregivers with diverse backgrounds. During member checking, participants were invited to clarify and verify the researchers’ interpretations of their experiences when necessary. Data-source triangulation was achieved by comparing accounts from different caregivers to identify convergent and divergent perspectives. Confirmability was supported through peer debriefing and reflexive memos, allowing researchers to critically reflect on how their professional backgrounds and prior assumptions might influence interpretation. All interview transcripts, codes, and thematic categories were independently reviewed and confirmed by the second, third, and fifth authors, and coding discrepancies were resolved through consensus discussions to ensure findings remained grounded in participants’ accounts. Dependability was ensured by maintaining a clear audit trail documenting data collection and analytic procedures. Transferability was enhanced through maximum variation sampling across key characteristics, including region, educational level, economic status, relationship to the patient, and social class, together with the use of rich descriptions and verbatim quotations.

## 3 Results

A total of 20 interviews were conducted with the family caregivers of patients with schizophrenia. There were 12 females and 8 males, with a mean age of (60.60 ± 9.52) years. The patients had a mean age of (57.25 ± 12.30) years. Eleven participants resided in urban areas and nine in rural areas. Most caregivers had taken care of the patients for more than 10 years. Participant characteristics are shown in **Table 1**.

**Table 1:** Characteristics of caregivers and patients (N=20).

Characteristics	Caregivers (n)	Patients (n)
Gender		
Female	12	9
Male	8	11
Age (years)		
30-60	9	11
>60	11	9
Residence		
Urban	11	11
Rural	9	9
Employment status		
Unemployed	7	14
Employed	4	1
Retired	9	5
Educational level		
Junior high school	5	N/A
Senior high school and above	15	N/A
Relationship		
Parent	4	4
Spouse	9	9
Sibling	3	3
Offspring	4	4
Caregiving duration (years)		
<10	3	N/A
10-20	6	N/A
>20	11	N/A
Disease duration (years)		
<10	N/A	2
10-20	N/A	2
>20	N/A	16

The analysis generated five themes describing family caregivers’ involvement in supporting home-based recovery among patients with schizophrenia. These themes reflect caregivers’ evolving understanding of recovery, their routine caregiving practices, and the tensions they experienced in balancing patient autonomy, caregiving responsibilities, and concerns about future care. Themes and subthemes are presented in **Table 2**.

**Table 2:** Themes and subthemes of family caregivers’ involvement in home-based recovery.

Themes	Subthemes
Caregivers’ redefinition of recovery as stability rather than cure;	–
Routine recovery involvement in medication management and symptom monitoring	Medication management in everyday caregiving; Monitoring symptoms and response to early signs of relapse
Experienced tensions between the patient’s independence and relapse prevention	Challenges in promoting independent living skills; Barriers to participation in daily family activities; Communication difficulties between caregivers and patients; Ambivalent attitudes toward the patients’ social interaction
Bearing family obligation and personal strain in sustained caregiving involvement	–
Uncertainty in sustaining caregiving and the patients’ future stability	–

### 3.1 Theme 1: caregivers’ redefinition of recovery as stability rather than cure

Over time, many family caregivers described a gradual shift in how they understood recovery. In the early stages, caregivers described profound uncertainty surrounding recovery, often feeling unsure about how to support their family member. U10’s account illustrates how uncertainty about recovery became intertwined with both persistent worry and a strong sense of responsibility:

“At the beginning, I felt completely helpless. I worried about him all the time. I couldn’t stop thinking about what might happen next, and I wasn’t sure how to help him.” (U10).

For U10, uncertainty extended beyond concerns about the patient’s future to include uncertainty about their own role in supporting recovery. The experience of not knowing how to help, while simultaneously feeling responsible for helping, created a persistent sense of helplessness that shaped everyday life. U4 described a similar sense of helplessness, but emphasized how the unpredictability of symptoms contributed to feelings of losing control:

“It (schizophrenia) was terrifying. When her symptoms got worse, her behavior seemed so unpredictable that I never knew what to do. No matter what I did, I felt like I had lost control of the situation.” (U4).

Across accounts, uncertainty surrounding recovery concerned not only whether patients would improve, but also how caregivers could support recovery in meaningful ways. Through ongoing caregiving experiences, caregivers gradually came to understood schizophrenia as a long-term condition requiring continuous management rather than cure. Reflecting this shift, U10 explained:

“It (schizophrenia) is something you just have to live with for the rest of your life. There’s no cure for it. All you can really do is to manage it and try to keep things from getting worse.” (U10).

Recovery was no longer viewed as a return to “normal” life. Instead, avoiding deterioration and sustaining manageable daily life became more realistic and meaningful goals. As caregivers adjusted their expectations to the realities of long-term caregiving, stability gradually became the primary focus of recovery.

Guided by this stability-oriented understanding, caregivers’ everyday involvement in home-based recovery focused on maintaining a stable and supportive environment. They closely monitored subtle changes in mood, adjusted family interactions to minimize stress, and encouraged patients to remain engaged in daily activities. As U4 explained:

“First of all, she has to stay on her medication to keep things stable. But family support and care are just as important. As a family, we try to keep things calm and harmonious. My spouse and myself are on the same page about how we care for her. No matter what’s going on, we don’t argue in front of her, because even small things can trigger her relapse. We also take her out for walks to help her relax. When she’s in a good mood, things tend to stay steady.” (U4).

Similarly, U7 described responding promptly to subtle emotional changes:

“When I notice he’s quiet or not quite himself, I would ask what’s going on right away. Even if he keeps going on about it, I remind myself to stay patient and really listen, because he needs to get it out. If he bottles everything up, that’s when the symptoms tend to come back.” (U7).

These practices reflected more than vigilance towards relapse prevention. Through ongoing monitoring, adapting family interactions, and responding to subtle changes, caregivers sought to maintain a sense of order in lives shaped by uncertainty. Their efforts were directed towards protecting a hard-won yet fragile stability, preserving patients’ connection to everyday life, and sustaining family continuity. In the absence of a cure, these practices also enabled caregivers to enact their sense of responsibility and maintain a meaningful role in the recovery process.

### 3.2 Theme 2: routine recovery involvement in medication management and symptom monitoring

#### 3.2.1 Medication management in everyday caregiving

Medication management was gradually embedded in caregivers’ daily routines and was commonly understood as essential for maintaining stability and preventing relapse. Most caregivers took responsibilities in reminding patients to take medication, supervising adherence, or obtaining prescriptions. As one caregiver noted:

“Most of the time, my husband takes his medication on his own and usually keeps to the schedule. But when his symptoms start to fluctuate, I have to keep an eye on him and make sure he actually takes it. Our grandson helps pick up the medication, and every October he takes his grandfather to the hospital for some tests to check if the medicine is causing any side effects.” (U7).

However, understanding of continuous pharmacological treatment varied across caregivers. Some caregivers, particularly in rural areas, reported irregular medication use or discontinuation when medication was perceived as ineffective:

“She’s not taking the medication recently. I didn’t think it was helping much. Even when she was taking it, I still had to keep watching her all the time.”(R8).

Caregivers’ accounts also reflected limited knowledge of medication-related adverse effects and their management. As a result, even when potential side effects were recognized, caregivers were often uncertain about how to address them. Some caregivers continued to prioritize medication adherence, accepting or overlooking these effects in an effort to maintain symptom stability:

“Lately she’s been taking her medication regularly. But when her symptoms get worse, she sometimes refuses to take it. I’ve noticed that her memory seems to get worse after she takes the medicine. But she really can’t stop taking it, because if she does, her symptoms get worse.” (R3).

#### 3.2.2 Monitoring symptoms and response to early signs of relapse

Caregivers were closely involved in monitoring fluctuations in patients’ emotional and behavioral states, which were often interpreted as early signs of relapse. Some described noticing that symptoms tended to worsen during particular periods or circumstances, prompting them to pay closer attention to changes in the patient’s condition.

“Her symptoms tend to get worse when the season changes, especially in autumn. During that time, I try to take her out for walks more often so she can look around and enjoy the scenery. It usually lifts her mood, and after about a week things settle down.” (U4).

For U4, symptom deterioration did not appear to be experienced as entirely unpredictable. Seasonal transitions had become recognized as times when the patient was more vulnerable to symptom worsening. Through repeated caregiving experiences, he appeared to have developed a practical understanding of patterns in the illness, allowing him to anticipate periods of instability and take steps that he believed might help the patient regain equilibrium.

Others described responding to signs of instability by avoiding direct engagement and waiting for the situation to settle.

“When he loses his temper, I usually just don’t react. I go about my own business and let him be. I try to be patient, so most of the time I just let it go.” (R1).

For R1, responding to emotional outbursts appeared to involve withdrawal rather than active intervention. Choosing not to react reflected a tendency to avoid situations that might provoke further distress or conflict.

### 3.3 Theme 3: experienced tensions between the patient’s independence and relapse prevention

#### 3.3.1 Challenges in promoting independent living skills

Despite recognizing the importance of independent living skills for recovery, many caregivers described taking primary responsibility for everyday tasks and holding modest expectations regarding the patient’s ability to live independently. R1’s account illustrates this perspective:

“He can take care of himself, but because of his illness, it’s hard for him to stick with things for very long. So, I try not to expect too much from him, because if I push too hard, it usually makes things harder for both of us.” (R1).

For R1, the patient’s difficulties were understood primarily through the lens of illness. Lowering expectations appeared to be a way of adapting to limitations that were perceived as largely beyond either the patient’s or caregiver’s control. Rather than focusing on developing new skills, recovery was understood in terms of accommodating the realities of the illness and avoiding frustration for both parties. In this sense, caregiving involved adjusting expectations to what felt realistically achievable.

In contrast, a few caregivers described actively encouraging patients to undertake everyday tasks such as household chores and personal care. U10 explained:

“He can manage the basics on his own, but I’m trying to help him become a bit more independent. I encourage him to do simple things like laundry or cooking, and most of the time he’s willing to give it a try.” (U10).

For U10, recovery involved more than maintaining stability. Encouraging activities such as cooking or laundry reflected a belief that the patient remained capable of developing greater independence despite the illness. These everyday tasks were valued not simply as practical skills, but as opportunities for the patient to participate more actively in daily life. In this sense, recovery was understood not only as symptom control, but also as supporting the patient’s capacity to function as independently as possible within the limits imposed by the illness.

#### 3.3.2 Barriers to participation in daily family activities

Caregivers often described involving patients in everyday family activities as part of supporting recovery. Activities such as sharing meals, watching television, and taking walks together were commonly viewed as achievable ways for patients to remain connected to family life. U7 explained:

“We usually have meals together and watch TV together, and he likes playing mahjong on his phone. I try to get him to do small things around the house, like washing the dishes. Even if he doesn’t do it very well, I still let him help a little—otherwise he just sits around. He rarely offers to help on his own, but if you ask him to do something, he’ll usually do it.” (U7).

For U7, participation appeared to be valued for the opportunity it provided the patient to remain involved in everyday life, rather than for the successful completion of tasks. Encouraging small contributions seemed to be a way of keeping the patient engaged in daily routines despite his limitations.

However, participation was often approached cautiously, with caregivers tending to lower expectations and avoid placing demands on patients. U3 reflected this protective approach:

“I try not to ask too much of him. If he feels like doing something, I just let him do it and mostly go along with him.” (U3).

For U3, supporting recovery appeared to involve protecting the patient from pressure rather than actively encouraging greater participation. Her account suggests that involvement in daily activities was largely left to the patient’s own initiative, with little expectation that he should take on additional responsibilities. While this approach may have helped preserve stability, it also appeared to limit opportunities for the patient to become more actively involved in everyday family life.

#### 3.3.3 Communication difficulties between caregivers and patients

Communication with patients was often described as a challenging aspect of home-based recovery. Through ongoing caregiving experiences, some caregivers gradually adapted their communication styles, becoming more cautious and patient in everyday interactions. Nevertheless, many continued to feel uncertain about how to communicate effectively when patients became emotionally distressed, resistant, or preoccupied with particular thoughts. R6 explained:

“It’s hard to communicate with her. She can be quite stubborn and tends to fixate on certain ideas. I’m usually a very straightforward person, but now I have to be careful about what I say—otherwise it might trigger her symptoms.” (R6).

For R6, communication appeared to involve a continual process of monitoring and adjusting responses. The contrast between being “straightforward” and needing to be “careful” suggests an ongoing tension between open expression and protecting his daughter from potential distress. Communication therefore became less a matter of expressing thoughts openly and more a process of judging how best to respond in ways that were both supportive and safe.

#### 3.3.4 Ambivalent attitudes toward the patients’ social interaction

Caregivers often expressed ambivalent attitudes toward patients’ social interaction beyond the household. While some recognized that maintaining contact with neighbors or relatives could support recovery, concerns about stigma, emotional overstimulation, or symptom relapse frequently made them cautious about broader social participation.

“When people say hello to him, I usually remind him to say hello back, and most of the time he does. He used to play cards with some of the older men in the neighborhood. But if someone says something unpleasant, he won’t argue—he just keeps it to himself, and I worry that might make his condition worse. After a few bad experiences, he stopped playing, and honestly, I’d rather he doesn’t start again.” (U7).

For U7, concerns about the potential consequences of negative social encounters appeared to influence how social participation was supported and encouraged. Maintaining stability seemed to take priority when opportunities for social engagement were perceived as carrying potential risks.

Several caregivers also expressed difficulty about how to support patients’ social engagement, particularly when patients tended to withdraw socially. While they often recognized the value of social participation, they frequently struggled to identify ways of encouraging engagement beyond the immediate family. U4 reflected:

“I’d like her to spend more time around other people, but she doesn’t really want to talk to the neighbors. If I ask her to say hello, she’ll just turn away and walk off. When relatives come over, she can chat with them at home, but going out to eat together is a different story. I think she might worry that people will look down on her.” (U4).

For U4, supporting social participation appeared to involve trying to understand the reasons underlying the patient’s reluctance to engage with others. Rather than attributing withdrawal solely to the illness, it was understood in relation to concerns about being judged or looked down upon by others. This understanding suggests an awareness that anticipated negative reactions from others could further diminish the patient’s willingness to engage in social interactions, while also highlighting the challenges caregivers faced in supporting participation beyond the family environment.

### 3.4 Theme 4: bearing family obligation and personal strain in sustained caregiving involvement

Sustained involvement in home-based recovery imposed considerable strain on family caregivers, particularly as they attempted to balance the needs of the patient with other family responsibilities. U11’s account illustrates this tension:

“When my mother becomes unwell, she says things that don’t make sense and can be really hurtful. If I try to get her to do something she doesn’t want to do, like taking a shower, she becomes very resistant and takes it out on me. It’s exhausting. The hardest part is feeling torn between caring for my mother and caring for my son. He’s in his second year of high school, and I often feel guilty that I can’t be there for him when he needs me most. The rest of the family don’t really understand, and they can’t help much with her care.” (U11).

For U11, caregiving involved more than responding to her mother’s symptoms; it meant navigating competing responsibilities that often left her feeling unable to fully meet the needs of either generation. What appeared particularly distressing was the persistent tension of being unable to fulfil important family roles simultaneously, as caring for one family member often came at the perceived expense of another. The limited understanding and involvement of other family members seemed to further intensify this burden, reinforcing a sense that responsibility for balancing the competing demands of care and family life rested largely with her.

Yet despite these tensions, U11 framed caregiving as a responsibility that could not be questioned:

“She’s my mother—it’s my responsibility. I try to keep things simple. What’s done is done, and there’s not much I can change. Over time, I’ve just learned to accept it and get on with it.” (U11).

For U11, accepting and “getting on with it” appeared to be a way of making sense of circumstances she felt unable to change. By defining caregiving as an unquestionable familial responsibility, she was able to sustain her involvement despite ongoing strain. At the same time, this understanding left limited space for acknowledging her own needs or questioning the personal cost of her involvement.

This tension between familial obligation and self-preservation was particularly evident among older caregivers. Declining health further intensified their physical exhaustion and psychological strain.

“Lately, I feel like my health isn’t what it used to be, and sometimes I just don’t have the energy. I also tend to worry a lot, so it can be quite draining mentally. Luckily, I have a few close friends I can talk to, and that really helps.” (U7).

Across accounts, caregivers commonly understood caregiving as a moral obligation shaped by cultural expectations of filial piety and parental duty within the Chinese context. These values provided meaning and motivation for sustained involvement in care, but also made it difficult for caregivers to prioritize their own wellbeing. As a result, many came to regard personal sacrifice and emotional distress as an expected part of fulfilling family responsibilities.

### 3.5 Theme 5: uncertainty in sustaining caregiving and the patients’ future stability

Uncertainty regarding the long-term continuity of caregiving and patients’ future stability emerged as a significant concern among family caregivers. As caregiving continued, many became increasingly aware that the stability they had worked hard to maintain remained closely tied to their own ongoing involvement. R2’s account illustrates how these concerns emerged through everyday caregiving experiences:

“If I go and stay with relatives for a couple of days, he’ll usually call the next day asking me to come home. He can’t really manage without me. My children would help look after him, but I worry they might not have the patience to care for him the way I do.” (R2).

For R2, years of caregiving had fostered a sense that her husband’s stability was closely linked to her continued presence. Through daily interactions and accumulated experience, she had developed a nuanced understanding of his needs and came to view caregiving as a responsibility that could not easily be transferred to others. The prospect of relying on family members therefore raised concerns not only about who would provide care, but also about whether the patient would receive the same level of understanding and responsiveness.

Reflecting on her own ageing made these concerns increasingly difficult to avoid:

“Sometimes I worry about what would happen to him if I passed away first. For now, as long as I’m able to look after him, I will. But if I can’t anymore, we’d probably have to consider a care facility.” (R2)

For R2, imagining a future in which she could no longer provide care meant confronting the possibility that the sense of stability they had built together might not endure. Her concerns suggest that she understood recovery not as an individual achievement, but as something sustained through an ongoing caregiving relationship. The prospect of her absence therefore represented more than the loss of practical support; it threatened the continuity of the routines, relationships, and shared understandings that had come to underpin her husband’s everyday life.

When caregivers considered a future in which they could no longer provide care themselves, institutional care was often viewed as the last resort to ensure basic supervision and daily care instead of promoting meaningful recovery. As U2 reflected:

“As long as I’m able to take care of him, I’ll keep doing it. If I can’t anymore, he’ll probably have to move into a care facility. I just hope there will be some government support—otherwise, I’m not sure we could afford it.” (U2).

U2’s account highlights how decisions about future care were shaped not only by concerns about continuity of support, but also by worries about affordability and the perceived limitations of institutional care in maintaining the patient’s quality of life.

Across accounts, discussions about future care and recovery were often limited, and proactive care planning involving patients was uncommon. Rather than reflecting a lack of awareness, this reluctance appeared to stem from the emotional difficulty of imagining a future in which caregivers could no longer fulfil a role that had become central to sustaining the patient’s stability.

For many caregivers, maintaining stability in the present became both a way of managing future uncertainty and a pragmatic response to limited care options. Their focus on everyday caregiving reflected an understanding that recovery was fragile and sustained through family relationships and daily routines. Consequently, concerns about the future extended beyond who would provide care to whether the patient’s hard-won stability could endure in their absence.

## 4 Discussion

This qualitative study explored the involvement of family caregivers in supporting home-based recovery among patients with schizophrenia in China, highlighting the challenges they encountered and the adaptive strategies families developed within the existing mental health system. Caregivers commonly reframed recovery as maintaining symptom stability rather than achieving cure. Monitoring medication adherence and responding to symptom fluctuations consequently became their routine caregiving practices. At the same time, caregivers experienced ongoing tensions between promoting patients’ functional recovery—particularly in relation to independent living, family life, family communication, and social interaction—and ensuring symptom stability. They also struggled to care for the patient while looking after their own physical and emotional wellbeing. Uncertainty in sustaining caregiving and ensuring patients’ future stability further reflected limited planning and support for future caregiving arrangements.

Caregivers’ perceptions of recovery strongly shaped their approaches to daily care. The study suggests recovery was primarily understood as maintaining stability and preventing relapse, leading caregivers to focus mainly on symptom monitoring and medication supervision rather than broader psychosocial recovery. However, many caregivers reported uncertainty in recognizing relapse warning signs and preventing relapse (47, 48). Evidence from China indicated insufficient mental health literacy among caregivers, with only 18.5% of caregivers aware of schizophrenia symptoms and merely 9.1% knowledgeable about relapse prevention (33). Difficulties in medication management further reflected limited mental health literacy among caregivers. Although caregivers generally assumed primary responsibility for supervising medication, many reported limited knowledge regarding medication management, side-effect responses, and treatment adjustment (22, 32–34, 49). In this study, these gaps were more pronounced in rural areas. Some caregivers also relied on medication discontinuation or switching rather than seeking timely professional support when side effects occurred (50). Together, these findings suggest that strengthening caregivers’ recovery-oriented knowledge may be important for promoting broader psychosocial recovery in home-based schizophrenia care.

Although recovery-oriented care emphasizes autonomy and meaningful living rather than symptom control alone (2), caregivers in this study often struggled to balance patients’ independence with concerns about symptom instability. Schizophrenia-related impairments in independent living, family communication, and social participation reinforced caregivers’ perceptions that continuous supervision was necessary (51). As a result, many caregivers adopted cautious and protective caregiving approaches intended to minimize stress and reduce relapse risk. However, such practices may inadvertently restrict opportunities for functional recovery. Insufficient understanding of schizophrenia and recovery processes further contributed to caregivers’ fear of symptom recurrence and reduced confidence in supporting recovery-oriented practices (28, 33, 52). This tension was particularly evident among parents and partners of patients with shorter illness duration, who expressed stronger needs for guidance regarding communication, relapse prevention, and recovery support. By contrast, prolonged caregiving appeared to foster adaptation but may also reinforce entrenched caregiving patterns and reduce receptiveness toward recovery-oriented concepts or educational interventions (22). Supporting caregivers in balancing symptom management with patients’ independence may therefore represent a key challenge for recovery-oriented community mental health services. Support strategies should also be tailored to caregivers’ needs and stages of caregiving.

Our findings revealed notable urban–rural disparities in caregivers’ capacity to support home-based recovery. Urban caregivers more frequently encouraged participation in household tasks and independent living skills, whereas rural caregivers tended to assume responsibility for daily activities to maintain stability and reduce perceived risks. These difficulties may reflect regional disparities in community mental health resources, caregiver education, and access to professional support (53, 54), suggesting that recovery-oriented caregiving practices are shaped by broader service inequalities. Community-based support strategies should therefore be adapted to local service conditions and caregiving contexts rather than implemented as uniform models.

Family involvement in home-based recovery remains insufficiently integrated into routine community mental health services (23). In China, current community-based mental health management is primarily embedded within public health services, where routine follow-up care largely emphasizes medication adherence and symptom surveillance over psychosocial recovery and family empowerment (55). In addition, family interventions are rarely incorporated into standard community mental health practice, further limiting external support for families of patients with schizophrenia (15, 16). These service limitations may further reinforce symptom-focused caregiving and reduce opportunities for recovery-oriented family support in the community.

Caregivers in this study experienced persistent tension between strong familial responsibility and maintaining their own wellbeing. Within the Chinese cultural context, caregiving for relatives with schizophrenia is commonly viewed as a moral and familial obligation rather than an individual choice (34). Caregiver well-being can affect the quality and sustainability of care (56, 57). Nevertheless, prolonged caregiving often places substantial physical, psychological, and social strain on caregivers (34, 58, 59). Limited emotional support from friends, relatives, and formal services further intensified feelings of exhaustion and isolation. Limited opportunities for social participation further constrained emotional relief and stress management (58, 60). Instead, caregivers often relied on personal endurance, self-adjustment, and cognitive reframing strategies, such as accepting schizophrenia as a chronic condition and lowering expectations regarding recovery (22). These findings suggest that caregiving burden in schizophrenia extends beyond practical tasks and is deeply shaped by cultural expectations surrounding family responsibility and self-sacrifice. Greater attention to caregivers’ emotional wellbeing and long-term support needs may therefore be necessary within community mental health services.

Future care for patients with schizophrenia were characterized by considerable uncertainty and limited long-term planning among caregivers. Care arrangements largely relied on family caregiving or public institutional care, with patients rarely involved in decision-making. Although some caregivers expected other family members to assume future caregiving responsibilities, many expressed uncertainty regarding the sustainability of such arrangements (61). Institutional care was generally viewed as the last-resort option when family caregiving became unmanageable and financial resources permitted (58). Notably, caregivers often prioritized maintaining present stability over discussing future planning, due to limited care options and ongoing caregiving demands. These findings highlight the lack of structured long-term care planning within current community mental health services and the limited availability of appropriate residential and social care resources for people with schizophrenia. Earlier and more structured family-centered future care planning may therefore be important in supporting long-term community living for people with schizophrenia.

To strengthen recovery-oriented family support within community mental health services, CHSCs should move beyond routine symptom surveillance toward family-focused psychosocial rehabilitation support. Given that caregivers often prioritized symptom stability over broader psychosocial recovery, routine follow-up visits could incorporate practical guidance on communication, patients’ independence, and community participation. Expanding such support will require greater workforce capacity, targeted training, and appropriate incentive mechanisms to enable community providers to address families’ psychosocial and rehabilitation needs. Support strategies should also be tailored to caregiving stage and local service contexts. Newly diagnosed families may benefit from psychoeducation and emotional support, whereas long-term caregiving families may require interventions addressing caregiver burden, social isolation, and patients’ functional recovery. Closer integration of specialized family interventions with community-based care may further enhance the provision of sustained guidance and support throughout the recovery process. Given the urban–rural disparities identified in this study, flexible community-based support models are particularly needed in under-resourced areas where access to psychiatric and rehabilitation services remains inadequate. Integrating caregiver burden assessment, peer-support programs, respite services, and future care planning into routine community mental health services may further strengthen the sustainability of long-term home-based care and promote recovery-oriented community living for people with schizophrenia.

Key strengths of this study include its in-depth qualitative exploration of family caregivers’ experiences in supporting home-based recovery among patients with schizophrenia. By including caregivers from both urban and rural communities, the study captures the complex realities of family-based care and the challenges caregivers face in balancing patients’ autonomy and symptom stability across diverse caregiving contexts. Participant quotations effectively support the identified themes and strengthen the credibility of the findings. In addition, the study is highly relevant to community mental health practice and offers meaningful implications for developing family-centered mental health services and strengthening community and institutional support for home-based recovery.

This study has several limitations. First, the sample size was relatively small, and participants were recruited from selected urban and rural districts in Beijing, which may limit the breadth and representativeness of the findings. In addition, voluntary participation may have introduced selection bias, as caregivers who were more willing to participate in the study may also have been more actively involved in patients’ recovery. Second, the study included only family caregivers and did not incorporate the perspectives of individuals with schizophrenia. As themes such as autonomy, social participation, and future planning are closely linked to patient’s lived experiences, the absence of patient perspectives may limit the interpretive depth of the findings. Future research should integrate both caregiver and patient perspectives to provide a more comprehensive understanding of home-based recovery. In addition, the transferability of the findings may be constrained by sociocultural differences and variations in healthcare systems across countries. Nevertheless, the insights generated from this study may still be relevant to caregiving contexts that share similar sociocultural characteristics and primary healthcare structures.

## 5 Conclusion

This study highlights the central role of family caregivers in supporting home-based recovery of patients with schizophrenia. Caregivers primarily prioritized symptom stability and medication adherence. However, supporting patients’ independence and maintaining their own wellbeing remained ongoing challenges. Recovery-oriented caregiving was further constrained by insufficient mental health literacy, insufficient community support, and disparities in community mental health resources. The findings underscore the need to strengthen recovery-oriented family support within community mental health services, particularly in promoting patients’ daily functioning, participation in family activities, family communication, and social interaction. CHSCs should make greater use of routine follow-up care to provide accessible psychoeducation, relapse prevention support, and practical rehabilitation guidance for family caregivers, with greater emphasis on patients’ functional recovery and social participation. Support should also be tailored to families’ caregiving stages and needs to strengthen caregivers’ confidence in supporting long-term home-based recovery.

## Data Availability

The original contributions presented in the study are included in the article/**Supplementary Material**. Further inquiries can be directed to the corresponding authors.
