# Supplementary material for: Family caregivers’ involvement in home-based recovery for patients with schizophrenia: a qualitative study in Beijing, China
Source: Front Psychiatry. 2026 Jul 13;17:1853599. doi: 10.3389/fpsyt.2026.1853599 (PMC13402536; doi:10.3389/fpsyt.2026.1853599)
Supplement: Supplementary file 1 [file Supplementaryfile1.docx]

***Supplementary Material***

1. **Interview Schedule**

**1.1　Introduction**

Thank you for taking the time to participate in this interview. This study is conducted by researchers from the School of General Practice and Continuing Education at Capital Medical University. As you may know, many individuals living with schizophrenia undergo recovery at home. This interview aims to gain a deeper understanding of your experiences as a family caregiver in supporting home-based recovery for your relatives. In particular, we aim to explore the challenges you encounter, as well as the adaptive strategies you develop within the primary healthcare system.

**1.2　Informed consent**

With your permission, we will audio-record the interview and take written notes throughout the conversation. All personal information collected will be kept strictly confidential and used exclusively for research purposes. If you agree to participate, please sign the informed consent form.

**1.3　Topic guide**

First, we will collect basic demographic information about you and your relative, along with detailed information on the patient’s symptoms, current clinical status, and other relevant clinical characteristics.

-How do you understand your relative’s mental illness and your involvement in supporting their recovery at home?

-How are you involved in managing your relative’s medication in daily life? What strategies do you use, and what challenges do you still face?

-When your relative’s symptoms recur or fluctuate, what strategies have you developed to manage and prevent these situations, and what challenges remain?

-How well can your relative manage daily life?What training or support have you provided to help improve their daily living skills, and what challenges do you still face?

-How does your relative usually interact with other family members in daily life? What efforts have you made to support or improve these interactions, and what challenges do you still face?

-What kinds of communication do you usually have with your relative? What approaches do you find effective for better communication, and what challenges do you face?

-Is your relative open to interacting with relatives or neighbors? What ways have you found that work well to encourage these interactions, and what challenges remain?

-As the primary caregiver, how would you describe your caregiving responsibilities and their impact on you? What kinds of stress or pressure do you experience, and how do you deal with them?

-Have you discussed any plans with your relative for their future care and recovery? What are your main concerns, and what kinds of support do you think would be needed?
